# NKG2D Ligands in Tumor Immunity: Two Sides of a Coin

**DOI:** 10.3389/fimmu.2015.00097

**Published:** 2015-03-04

**Authors:** Jinyu Zhang, Fahmin Basher, Jennifer D. Wu

**Affiliations:** ^1^Department of Microbiology and Immunology, Medical University of South Carolina, Charleston, SC, USA; ^2^Cancer Immunology Program, Hollings Cancer Center, Charleston, SC, USA

**Keywords:** NKG2D ligands, cancer, NK cells, T cells, tumor immunity

## Abstract

The activating/co-stimulatory receptor NKG2D (natural-killer group 2, member D) is expressed on the surface of all human NK, NKT, CD8^+^ T, and subsets of γδ^+^ T cells. The significance of NKG2D function in tumor immunity has been well demonstrated in experimental animal models. However, the role of human NKG2D ligands in regulating tumor immunity and cancer prognosis had been controversial in the literature. In this review, we summarize the latest advancement, discuss the controversies, and present evidence that membrane-bound and soluble NKG2D ligands oppositely regulate tumor immunity. We also discuss new perspectives of targeting NKG2D ligands for cancer immunotherapy.

## Introduction

The discovery of the natural killer (NK) cell activating receptor NKG2D and its ligand MHC I chain-related molecule (MIC) expression on human cancer cells marks one of the most significant milestones in understanding NK cells in cancer immune surveillance in the last several decades (1–4). NKG2D, initially identified on NK cells, was also found to be expressed by CD8 T cells (only activated CD8 T cells in mouse), NKT cells, and subsets of γδ T cells (4–6). *In vitro* studies have demonstrated that ligation of NKG2D by its ligands provides activating signal to NK cells and co-stimulatory signal to T cells (5, 7, 8). *In vivo* studies with experimental animals have demonstrated that ectopic expression of NKG2D ligands on tumor cells is sufficient to cause tumor rejection (9, 10). These data suggested that sustaining or increasing NKG2D expression on tumor cells should be a viable avenue for developing effective cancer immunotherapy. However, human cancer cells are found to broadly express NKG2D ligands and yet progress (1, 11). Moreover, clinical studies with different cancer types and even different disease stages within the same type of cancer presented conflicting prognostic values of NKG2D ligands (12–15). The conundrum and controversy have withheld the further exploration of NKG2D ligand-mediated cancer immunotherapy. In this review, we will analyze controversial clinical studies and consolidates the discrepancy in the literature. We will present most updated evidence that different physical forms of NKG2D ligands could oppositely regulate tumor immunity. We also propose therapeutic perspectives and options for exploiting NKG2D ligands-based cancer immunotherapy. Finally, we will also introduce a newly developed pre-clinical “humanized” mouse model for validating immunotherapies to target NKG2D ligands.

## NKG2D, an Activating Immune Receptor

Natural-killer group 2, member D, also known as Klrk1 (NKG2D), a C-type lectin-like receptor, was firstly identified in natural killer (NK) as an activating immune receptor (16). In human, NKG2D is not only expressed by all NK cells (17, 18), but is also expressed by all CD8^+^ T cells, and subsets of γδ^+^ T cells as a co-stimulatory receptor (2, 6, 8, 19). In mice, NKG2D is also expressed by all NK cells, however, only by activated CD8^+^ T cells and approximately 25% of spleen γδ^+^ T cells (7). NKG2D is also expressed by activated mouse macrophage (7). NKG2D is rarely expressed by mouse intestinal intraepithelial γδ^+^ T cells (20). Little or no CD4^+^ T cells naturally express NKG2D in both human and mouse. Notably, NKG2D is not only expressed in mice and humans but also expressed in other mammals (21, 22).

**Figure 1:**
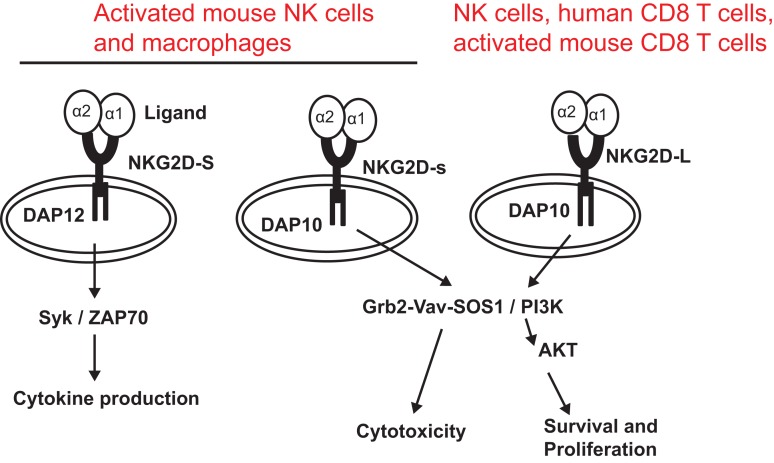
**NKG2D receptor and signaling pathways associated with adaptor molecules**. The NKG2D receptor forms a homodimer that associates with the adaptor molecules DAP10 or DAP12. Association of NKG2D with DAP10 initiates PI3K and Grb2–Vav–Sos1 signaling pathways, whereas association of NKG2D with DAP12 initiates Syk/ZAP70 signaling cascade. NKG2D-S can promiscuously associate with DAP10 or DAP12. NKG2D-L primarily associates with DAP 10. Whether NKG2D-L also associates with DAP12 is controversial. NKG2D-L is expressed by all NK cells, human CD8 T cells, and activated mouse CD8 T cells. Activated mouse NK cells and macrophages express NKG2D-S. Whether activated human NK cells and CD8 T cells express NKG2D-S is also controversial.

NKG2D is a type II transmembrane glycoprotein, which does not contain any known signaling elements in the intracellular domain (23). Resembling many activating receptors, NKG2D depends on an adaptor molecule to initiate signaling transduction and cellular activation. Two adaptor molecules, DNAX-activating proteins of 10 kDa (DAP10) and 12 kDa (DAP12), were identified to associate with NKG2D. The selection of adaptor molecule association depends on the cell types and the isoforms of NKG2D. In resting mouse NK cells, NKG2D exclusively associates with DAP10; whereas in activated NK cells, NKG2D associates with DAP10 and DAP12 (24, 25). In both human and activated mouse T cells, NKG2D exclusively associates with DAP10, presumably due to CD8 T cells lacking DAP12 expression (20, 23). In mice, alternative splicing generates two NKG2D isoforms, NKG2D long (NKG2D-L) and the alternatively spliced NKG2D short (NKG2D-S) which is 13 amino acid shorter in the cytoplasmic N-terminus than NKG2D-L (25). Initial study with primary mouse NK cells demonstrated that NKG2D-S associated with DAP10 or DAP12, whereas NKG2D-L exclusively associated with DAP10 (25). A more recent study found that DAP10 and DAP12 competed equally for binding NKG2D variants when co-expressed in CD8 T cells (26). These findings suggested that NKG2D signaling could be more complicated than our current understandings. Similarly, the isoform NKG2D-s was found initially only in activated mouse NK cells and later was also described in naïve mouse NK cells (25). Whether activated human NK cells and macrophages also express NKG2D is controversial to date due to discrepancies in different experimental settings by different investigators (27, 28). DAP 10 and DAP12 association activates different signaling pathways (Figure 1). The DAP10 molecule contains an YXXM tyrosine-based motif (23) that recruits the p85 subunit of phosphoinositide kinase-3 (PI3K) and growth factor receptor-bound protein 2 (Grb2). This signaling cascade is similar to which delivered by the T cell co-stimulatory molecules CD28 and ICOS (29, 30). The DAP12 molecule contains an ITAM, which recruits ZAP70 and Syk to mediate NK cell activation (25). The selection of adaptor molecule and the ensuing activation of different signaling pathways may explain the functional differences of NKG2D in NK cells and T cells. Binding of NKG2D to its ligands is sufficient to induce NK cell activation, even with inhibitory signals, such as MHC molecules (31), but only co-stimulates CD8 T cells or γδ T cells (8, 32).

NKG2D expression and signaling can be regulated by cytokines and tumor-derived factors. Cytokines, such as IL-2, IL-7, IL-12, IL-15, and type I interferons (IFNs) increase cell surface expression of NKG2D (33). IL-15 signaling not only regulates NKG2D expression but also increases the expression of DAP10 and phosphorylates the adaptor molecule to prime NKG2D signaling (34). On the contrary, cytokines such as IL-21, IFN-γ, and TGF-β have been shown to decrease NKG2D expression. IL-21 has been reported to reduce expressions of DAP10 and NKG2D in human CD8^+^ T cells and NK cells (35). In mice, IL-21 stimulation of NK cells is dependent on regulating the NKG2D expression in mouse model of breast carcinoma (36). TGF-β has been shown to downregulate NKG2D expression; however, the effect can be reversed by IL-2 or IL-18 (37).

## Regulation of NKG2D Ligands

**Figure 2:**
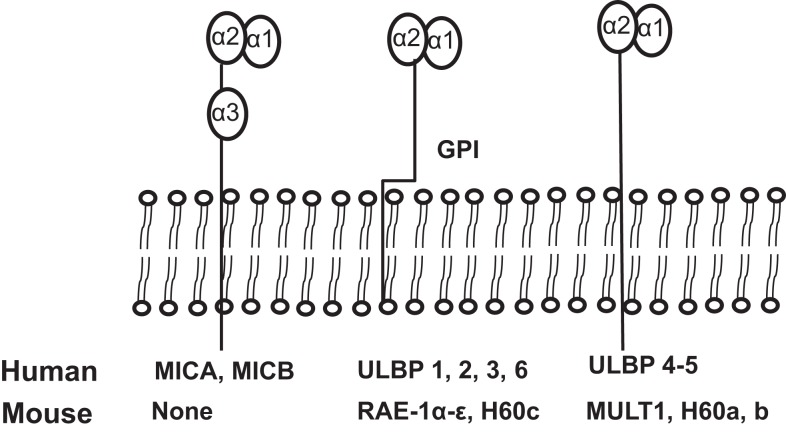
**Schematic illustration of identified ligands for human and mouse NKG2D**. The unique human NKG2D ligands MICA and MICB have α1, α2, and α3 extracellular domains and transmembrane domains, sharing structural homology with MHCI. All other human NKG2D ligands and all mouse NKG2D ligands lack the α3 domain. Murine NKG2D ligands, RAE1 α–ε and H60c, and human ULBP1-6 are glycosylphosphatidylinositol (GPI)-linked to the membrane. Human ligands, ULBP4 and ULBP5, and mouse ligands, MULT1, and H60a and H60b, are transmembrane proteins.

NKG2D ligands are structural homologs of MHC class I molecules. NKG2D ligands are absent or rarely expressed in normal tissues, but are extensively expressed in various malignancies and viral-infected tissues (8, 11). The identified human NKG2D ligands include class-I-related chains-related molecules A and B (MICA and MICB) proteins and retinoic acid early transcripts-1 (RAET1), also known as UL-16 binding proteins (38). To date, identified mouse NKG2D ligands include five different RAET1 isoforms (RAET1α, RAET1 β, RAET1 γ, RAET1 δ, and RAET1 ε), three different H60 isoforms (H60a, b, and c), and one MULT1 gene (39, 40) (Figure 2). No MIC homologs have been identified in rodents. Although NKG2D ligands are structural homologs of MHC class I molecules, they do not present antigen to T cells or bind β2-microglobulin (1).

NKG2D ligands are prevalently expressed in human malignancies. MIC and ULBP families of ligands are found prevalently expressed by carcinomas of the breast, lung, colon, ovary, kidney, and prostate, melanomas, gliomas, and leukemia (2, 11, 12, 41–43). Certain normal human tissues such as gastric and bronchial epithelia were found to express NKG2D ligand MIC (44). Given that NKG2D ligand expression can be induced by stress and that gastric and bronchial mucosal epithelia are frequently exposed to microbial organisms (1), these observations are anticipated. The gut epithelium constitutively expresses MICA; however, the expression can be altered with changes in the gut flora in inflammatory states such as Crohn’s disease and celiac disease (45, 46). Of note, NKG2D ligands are also found to be upregulated in activated T cells of both human and mouse origin (47, 48). Collectively, these studies suggest that NKG2D ligand expression in tissues is regulated by the pathogenic physiological environment to adequately differentiate between healthy and potential target cells.

Regulation of NKG2D ligand exerts at different levels, including transcription, mRNA stability, translation, protein stabilization, and excretion/shedding of ligands from cells (40, 49–51). DNA damage response induces the transcription of an array of NKG2D ligands, such as *ULBP1* and *ULBP2* and MIC family molecules (52). Regulation of NKG2D ligands in response to DNA damage depends on the accumulation of cytosolic DNA and the induction of nucleotide sensing pathways, which activate the TANK-binding kinase 1 and the IFN regulatory factor 3 (53). In addition, *MICA*, *MICB*, and *ULBP3* transcripts have been reported to be targeted by endogenous microRNAs (54). Inhibition of PI3K impaired *Raet1* transcript levels, and was further observed at the protein level suggesting that PI3K regulates *Raet1* at the transcriptional and post-transcriptional stage (55). In addition to its role in transcriptional regulation of *MICA* and *MICB* (56), heat shock counteracts ubiquitin-mediated degradation of MULT1 (57). At the post-translational level, NKG2D ligands such MICA, MICB, and ULBPs were found to be cleaved from the cell surface by proteases ADAM10/17 and MMP14 and in some cases by tumor-derived exosomes (58–62). Notably, expression of NKG2D ligands was also shown to be downregulated by cytokines such as IFN-γ, IFN-α, transforming growth factor-β, and IL-10, although the underlying mechanisms have not been elucidated extensively (63).

## Two Faces of NKG2D Ligands in Regulating Tumor Immunity

Although induction of NKG2D ligand expression is associated with oncogenesis, expression of NKG2D ligands does not favor progression to carcinoma. In fact, multiple lines of evidence conclusively demonstrated that NKG2D ligand-induced receptor activation plays a significant role in tumor immune surveillance. Ectopic expression of NKG2D ligands on diverse tumor cell lines resulted in tumor rejection in experimental animal models through activation of NK cells and in some cases CD8 T cells (7, 9, 10, 52). To support the significance of ligand-engaged NKG2D function in tumor inhibition, an elegant study by Smyth et al. demonstrated that antibody neutralizing NKG2D increased the incidence of MCA-induced sarcoma formation (64). Furthermore, Guerra et al. using a genetic approach showed that NKG2D-deficient transgenic adenocarcinoma of the mouse prostate (TRAMP) developed three times more aggressive tumors than NKG2D-intact TRAMP littermates (65). Together, these studies in experimental animal models demonstrated that immunity mediated by ligand–NKG2D interaction plays a significant role in controlling tumor initiation and tumor progression.

In contrast to the conclusive data from animal models, clinical data eliciting the significance of NKG2D ligands in tumor immunity are rather controversial and presented discrepancies in conclusion. In early stages of breast and colorectal cancer, levels of tissue MIC expression were shown to positively correlate with improved survival (12, 15). In invasive breast and ovarian cancer, levels of tissue MIC expression were shown to correlate with poor prognoses (13, 14). These conflicting clinical observations are most likely resulted from discrepancies on how the ligand expression is assayed. Human cancer cells have broadly adapted a proteases or exosome-mediated strategy to shed surface NKG2D ligands (51). It has been proposed that membrane-bound NKG2D ligands stimulate immunity whereas soluble NKG2D ligands impair host immunity. The concept that membrane-bound and soluble forms of NKG2D ligands oppositely regulate tumor immunity was recently demonstrated by two elegant lines of “humanized” transgenic animals. Liu et al. created two lines of transgenic animals that express native human MICB and engineered shedding-resistant mutant MICB (MICB.A2) specifically in the mouse prostate under the control of prostate-specific promoter. These two lines of transgenic animals have no differences phenotypically. However, when they were crossed with the TRAMP mice that develop spontaneous prostate tumors, TRAMP/MICB mice exhibited expedited tumor progression in comparison to TRAMP littermates whereas TRAMP/MICB.A2 mice enjoyed tumor-free survival (66). Liu et al. further demonstrated that the tumor progression in TRAMP/MICB mice was due to elevated serum sMIC and severe impaired of host immunity, exemplified by depletion of peripheral NK cell populations. This study provided the first and strongest *in vivo* evidence that membrane-bound NKG2D ligands and soluble NKG2D ligands play opposite roles in tumor immunity. Given that tumor cells express more proteases as disease progression and that elevated levels of serum sMIC correlates with invasive malignancies in many types of cancers (66, 67–71), the conundrum of different clinical observation may not be “conflicting” after all if the assay can distinguish tissue levels of sMIC versus membrane-bound MIC.

Soluble NKG2D ligands suppress tumor immunity via multiple mechanisms. Downregulation of NKG2D expression on effector cells by soluble NKG2D ligands is the most common mechanism described thus far (2, 7, 9, 10, 64–66). Hanaoka et al. described that sMIC can also induce loss of the CD3ζ signaling adaptor, which occurs in T cells and NK cells in cancer patients, after NKG2D signaling leads to Fas-mediated caspases 3/7 activation and cleavage of CD3ζ (72), leading to T cell and NK cell dysfunction. Liu et al. have recently described that sMIC impairs the ability of NK cells to self-renew in tumor host and thus perturbs NK cell homeostasis (66). Given that NK cells play a significant role in shaping adaptive immunity by providing IFNγ and priming dendritic cells, the magnitude of negative imprints of sMIC on tumor immunity may be larger than we have understood. Nonetheless, these studies imply the critical role of NKG2D ligands in regulating tumor immunity although the mechanism may be a complex.

## Targeting NKG2D Ligands for Cancer Immunity

The significance of NKG2D-mediated immunity in controlling tumor development and progression has been well demonstrated. It is also evident that different membrane-bound and soluble NKG2D ligands oppositely regulate NKG2D-mediated tumor immunity. Based on these understandings, cancer therapeutic interventions should be focused on at least the following aspects: (a) sustain and enhance NKG2D expression and signaling on effector cells; (b) sustain and enhance the level of membrane-bound NKG2D ligand on tumor cells; (c) eliminate soluble NKG2D ligands.

**Figure 3:**
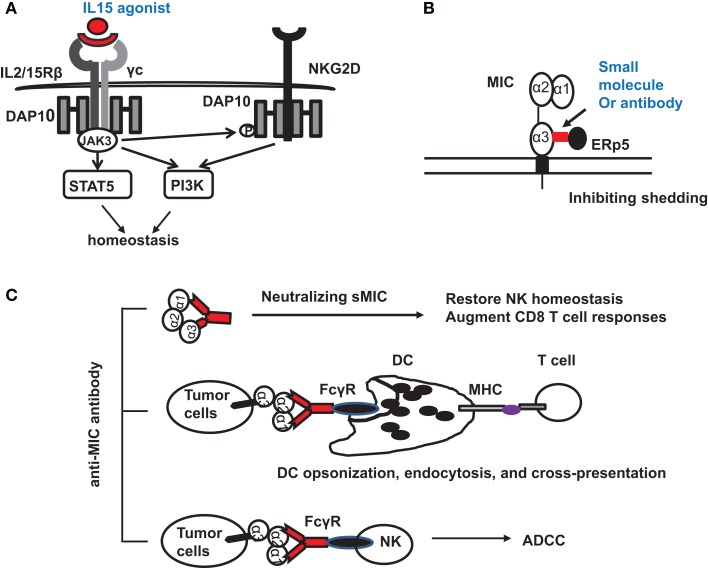
**Potential therapeutic interventions to target NKG2D ligand MIC for cancer immunotherapies**. **(A)** Counteracting the negative effect of soluble MIC on the receptor and NK cells with IL-15 agonists. Signaling initiated by IL-15 agonists not only results in JAK3-mediated activation of STAT5 and PI3K pathways to support NK cell proliferation and survival but also phosphorylates the adaptor DAP10 to prime NKG2D signaling. **(B)** Antibody or small molecule to block the interaction of MIC with ERp5 and thus inhibits MIC shedding. **(C)** Therapy with MIC specific antibody to: (i) neutralize serum sMIC and thus revive NK cell homeostasis and augment CD8 T cell function; (ii) opsonize tumor cells to enhance DC endocytosis through Fc receptor and cross-presentation of tumor antigen to effector T cells; (iii) induce antibody-dependent cytotoxicity of NK cells.

Tumor-derived sMIC has been shown to not only downregulate NKG2D expression on effector cells but also to perturb NK cell peripheral maintenance. One candidate strategy to overcome or reduce the negative effect of sMIC on effector cells is through intervention of IL-15 agonists. As a member of the gamma cytokine family, IL-15 can not only increase NKG2D expression (10, 73–75) but also cross-prime NKG2D signaling pathways (34). We have shown that IL-15 can restore and further augment NKG2D expression on NK cells isolated from cancer patients that have lost surface NKG2D expression (76, 77). Moreover, IL-15 has been shown to be important for NK cell homeostatic maintenance (78, 79) (Figure 3A). With IL-15 or its agonist currently in clinical trial, this therapy alone or in combination with strategies targeting sMIC is soundly plausible.

With the understanding of how shedding of MIC is regulated in tumor cells (49, 51), several therapeutic targets may be considered. Proteases, such as ADAM (a disintegrin and metalloproteinase)-10, ADAM-17 (60), and MMP-14 have been found to directly contribute to MIC shedding through proteolytic activities (62, 80). The thiol isomerase ERp5 has been shown to regulate MIC shedding by reducing the disulfide bond of MIC to induce conformational changes and thus allow the access of proteolytic enzymes (81). Theoretically, ERp5 and the MIC-shedding-contributing proteases should be considered as therapeutic targets. However, given the broad and constitutive function of these molecules in normal cellular physiology, therapeutic targeting could be challenging to avoid systemic toxicity. However, a highly conserved six-amino acid (6-aa) motif in the α3 domain of MIC has been identified to regulate the interaction of MIC with ERp5 and thus the shedding of MIC (82). Mutation of the six-amino acid motif completely prevented MIC shedding and enhanced tumor cell susceptibility to NK cell killing (82). Given the restricted expression of MIC, targeting the MIC-shedding regulatory motif may be more feasible for therapeutic intervention to sustain tumor cell surface MIC expression (Figure 3B).

One of the promising strategies to overcome immune suppressive effect of sNKG2D ligands is neutralizing antibody (Figure 3C). The idea is supported by clinical observations demonstrating that patients who developed anti-MIC autoantibody had reduced serum sMIC and better prognosis (83). In a clinical trial of melanoma patients with anti-CTLA-4 antibody blockade vaccine with autologous tumor cells engineered to express GM-CSF, it was shown that patients with high level of anti-MICA autoantibody presented better response to therapy (84). At least three mechanisms could contribute to the enhanced therapeutic responses. The first would be the evident reduction of serum sMIC to eliminate its immune suppressive effect. The second is through antibody opsonizing antigen presenting DC cells to enhance cross-presentation of tumor cells that express membrane-bound MIC (84, 85). The third may be through antibody-mediated ADCC effect to enhance NK cell killing of tumor cells that express membrane-bound MIC.

As a final reminder, the efficacy of targeting MIC-shedding motif or of an antibody directly targeting sMIC as novel intervention for cancer immunotherapy needs to be validated in pre-clinical models before moving to clinical trials. Due to the limitation that no human MIC homology is identified in rodents and that tumor shedding of MIC is only described in cancer patients, no clinically relevant animal model has been available to validate these therapeutic interventions until the recently described TRAMP/MIC double transgenic mouse model (66). Liu et al. described that the TRAMP/MIC mice closely resemble a broad spectrum of MIC^+^ cancer patients in the dynamic interaction of MIC-shedding and cancer progression. This model should provide a very useful tool for pre-clinical validation of targeting NKG2D ligand MIC for cancer immunotherapy.

## Conclusion

In summary, it is evident that NKG2D-mediated immunity is significant in potentiating anti-tumor responses. The exploitation of the NKG2D/NKG2D ligands pathway could be a promising strategy for anti-tumor immune therapy. However, NKG2D ligands present two contradictory faces in regulating NKG2D-mediated anti-tumor immunity. Membrane-bound NKG2D ligands stimulate anti-tumor immunity whereas soluble ligands suppress anti-tumor immunity. Based on these understandings, interventions for cancer immunotherapy should aim at enhancing NKG2D expression and signaling in effector cells, sustaining the highest levels of NKG2D ligands on tumor cell surface, and eliminating soluble NKG2D ligands. The recently developed “humanized” TRAMP/MIC mice should serve as a powerful tool for validating various targeting strategies for MIC^+^ malignancies, which represents the majority of human cancers.

## Conflict of Interest Statement

The authors declare that the research was conducted in the absence of any commercial or financial relationships that could be construed as a potential conflict of interest.
